# Colorimetry characteristics and color contribution of fluorescence in natural Cr-containing spinel

**DOI:** 10.1038/s41598-023-29675-w

**Published:** 2023-02-10

**Authors:** Jun Tang, Ying Guo, Jun Zhang

**Affiliations:** 1 School of Gemmology, China University of Geosciences (Beijing), Beijing, 100083 China; 2 National Gems & Jewelry Testing Co. Ltd, Beijing, 100013 China

**Keywords:** Mineralogy, Geochemistry

## Abstract

Fluorescence plays an important role in determining the color appearance of fluorescing minerals. This paper discusses the color mechanism in the red spinel and the color effects from the light source as well as the background on the spinel color. Excitation-Emission Matrices (EEMs) fluorescence spectroscopy was utilized to characterize the fluorescence of natural Cr-doped magnesia-alumina spinel from Myanmar. EMP, LA-ICP-MS, and optical spectroscopy were applied to study the spinel's chemical compositions and color mechanism. X-Rite Ci-7800 spectrophotometer, which is useful to measure colors for fluorescing minerals, was employed to acquire color data of spinel in daylight and incandescent light. The results indicated the higher value of Cr/Fe makes pinkish-red spinel has a much stronger red fluorescence effect than dark-red spinel. The two narrow absorption bands at ~ 25,500 cm^−1^ (with a broad absorption band at ~ 24,100 cm^−1^) and ~ 18,570 cm^−1^ in the optical absorption spectrum are assigned to spin-allowed electronic *d-d* transitions ^4^*A*_2*g*_ → ^4^*T*_1*g*_(F) and ^4^*A*_2*g*_ → ^4^*T*_2*g*_(F) in Cr^3+^ at the *M* site. The EEMs spectra of the pinkish-red spinels show twin emissions at 706 nm excited both by ~ 380 nm and ~ 535 nm radiations, which is the key to the bright neon red color in pinkish-red spinel. The colorimetry study suggests the strong red fluorescence produced by < 460 nm radiation contributes more than 10% lightness and chroma in pinkish-red spinel under daylight. The lightness and the chroma of the spinels grow with the decrease of the background grayscale. The differentiation of spinel colors in dark conditions is lower than that in a bright environment.

## Introduction

Spinel oxides, typically with the composition AB_2_O_4_ (where A atoms tetrahedrally coordinated (*T*) and B atoms reside in octahedral coordination (*M*)), form a large group of minerals and are frequently utilized as gemstones or ceramic pigments due to their wide range of intense colors^1^. The process that creates mineral color is vital for the reasons that color determines the value of a mineral, and it is one of the fundamental physical properties that is frequently applied to mineral identification^2^. Almost all of the main group metals and transition metals have been observed in spinel^3^, which makes spinel have a wide range of colors. The color of spinel is determined by the combined effects of two or more transition metals including Cr^3+^, Fe^2+^, Fe^3+^, Mn^2+^, Co^2+^, Cu^2+^, and V^3+^. The color mechanisms in spinel generally include electronic transitions involving the *d* orbitals of transition metal cation, and charge transfer between oxygen and ligand^4^.

Optical absorption spectroscopy refers to spectroscopic techniques that measure the absorption of radiation, as a function of frequency or wavelength, due to its interaction with a sample, which is useful for calculating the body color and measuring site occupancies and oxidation states of transition metals in mineral^5,6^. It has been used for decades to study the color of materials. Nonetheless, it’s imprecise to integrate the color by using optical spectroscopy for daylight fluorescing minerals because of the interferences from the fluorescence in themselves^7^. Fluorescence is the emission of light by a substance that has absorbed light or other electromagnetic radiation, and it is utilized as a valuable tool in mineralogical sciences. There are many researches about the significant effects of fluorescence on the color appearance in minerals in the past decades, such as diamond^8^, ruby^9^, opal^10^, amber^11^, and spinel et al. Natural gem-quality spinels are found all over the globe, such as Myanmar^12^, Vietnam^13–15^, Afghanistan^15^, Sri Lanka^14^, Tajikistan, and Tanzania^16^. According to the report by Vincent Pardieu, a special variety of natural spinel (typically use as “Jedi spinel” as a commercial name) with a bright neon pinkish-red color was found in Mogok and Mansin in Myanmar^12^. It is widely believed that the high-intensity red fluorescence in this kind of Cr-containing magnesium–aluminum spinel is the key to its unusual color^12,17^. Even though it has been proved that fluorescence of MgAl_2_O_4_: Cr^3+^ is mainly caused by Cr^3+^ ions in the strong octahedral crystal field by using photoluminescence (PL) spectroscopy^18^, however, the contribution of the strong red fluorescence on the color appearance of the spinel hasn’t been studied appropriately yet.

Furthermore, it should be noted that the perceived color of a transparent mineral is the combined effects of its selective absorption to light, thickness, or light path length (Beer-Lambert law), light sources^19^, and background colors^20^. To study the color of the pinkish-red spinel precisely, a functional modern color system is needed such as the CIE1976 L^*^a^*^b^*^ uniform color space system, which has been widely applied in exploring mineralogical colorimetry^21–23^. As mentioned above, the common way to calculate color does not apply to daylight-fluorescing minerals. However, the X-Rite Ci-7800 sphere benchtop spectrophotometer considers fluorescence and photoluminescence effects during color measuring and calculating^24–26^. It provides three UV filters (400 nm, 420 nm, and 460 nm) for color control and is useful for color research in fluorescing materials.

In this contribution, Excitation-Emission Matrices (EEMs) fluorescence spectroscopy was applied to the research about fluorescence in the pinkish-red spinels compared with that in dark-red ones. The color mechanism in the red spinel and the color effects from the light source and background were discussed. Towards this end, Laser Ablation Inductively Coupled Plasma Mass Spectrometry (LA-ICP-MS), Electron microprobe (EMP) analysis, optical spectroscopy, and sphere benchtop spectrophotometer were employed in this research.

## Results and discussions

### Chemical analysis

Spinel has a large group of varieties according to its composition. The chemical compositions of the spinel samples were measured using both EMP and LA-ICP-MS methods. The results are shown in Table 1, confirming the pinkish-red and the dark-red spinels all belong to the Mg(Al,Cr)_2_O_4_ series. Both methods show good agreement on the absolute concentrations of vanadium, chromium, iron, and manganese which act as chromophores in spinel. However, the chromium concentration is relatively higher than iron and other trace elements, which corresponds to the following discussions in optical absorption spectroscopy. It should be noted that the pinkish-red spinel has a higher value of Cr/Fe than the dark-red one, which makes it has a strong red fluorescence effect. Our focus here was to measure the concentrations of chromophore elements in spinel and so the analyses for other chemical contents in spinel are a subject for further study.

**Table 1:** EMP and LA-ICP-MS data for six studied spinel crystals.

	Color	Pinkish-red			Dark-red		
	Sample	SPI-01	SPI-02	SPI-03	SPI-04	SPI-05	SPI-06
EMP (wt%)	MgO	27.64	27.55	26.87	27.26	27.40	27.09
	Al_2_O_3_	71.53	71.99	71.14	70.98	71.27	70.38
EMP (ppmw)	V	nd	nd	nd	nd	nd	nd
	Cr	6876.32	5747.37	6007.37	10,509.47	6486.32	8265.26
	Fe	804.25	101.51	699.58	2076.99	1523.54	1679.00
	Mn	0.00	0.00	472.54	325.35	0.00	85.21
LA-ICP-MS (ppmw)	V	397.72	443.97	502.76	435.18	484.97	305.09
	Cr	7005.27	5813.98	6148.88	10,775.38	6312.81	8370.60
	Fe	757.37	117.81	730.30	2202.85	1527.63	1685.43
	Mn	3.36	12.70	490.84	317.86	10.75	85.31

*nd* not detected.

### Optical spectra

**Figure 1:**
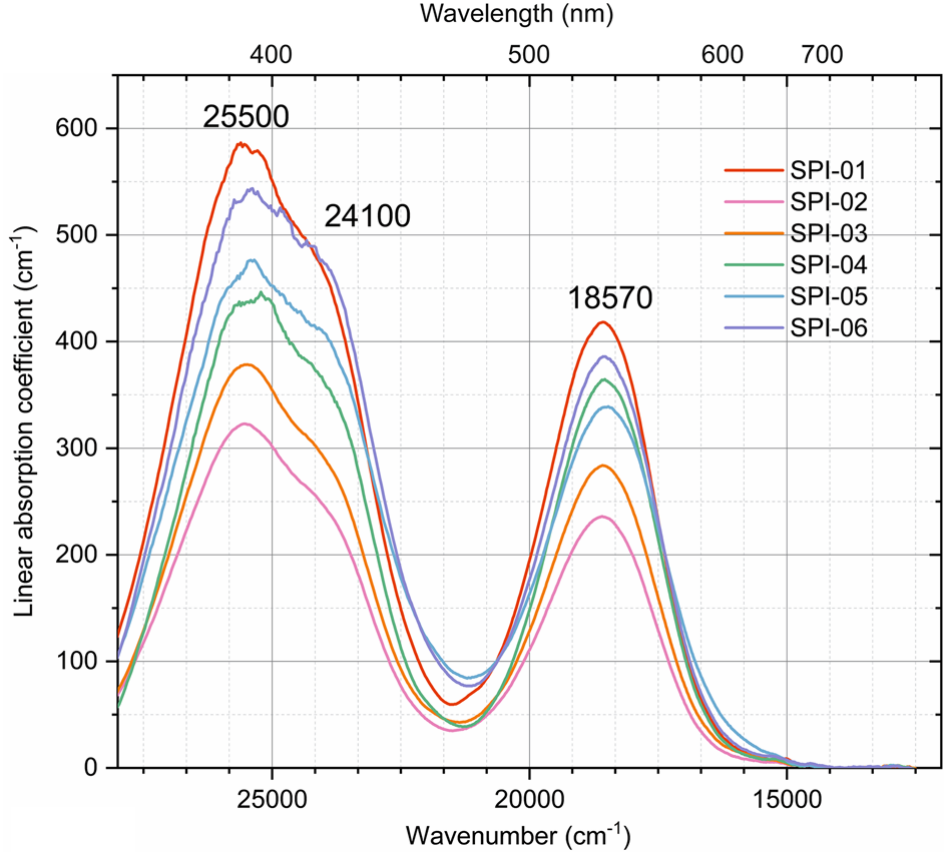
Optical absorption spectra of the present spinels.

The optical absorption spectra of the spinel crystals were measured and transferred into liner absorption coefficient for better comparison (Fig. 1). The recorded optical absorption spectra of the spinel crystals are in agreement with published spectra of natural Cr-containing spinels (e.g., Wood et al.^27^), showing two narrow absorption bands at ~ 25,500 cm^−1^ (with a broad absorption band at ~ 24,100 cm^−1^) and ~ 18,570 cm^−1^. The absorption bands of the spinel are assigned to spin-allowed electronic *d-d* transitions ^4^*A*_2*g*_ → ^4^*T*_1*g*_(F) and ^4^*A*_2*g*_ → ^4^*T*_2*g*_(F) in Cr^3+^ at the *M* site. The common way to calculate the color of the material is a complex integration that considers the optical absorption of the material itself, the relative spectral power distribution of the light source, and the color-matching functions of human eyes. Since the similar absorption shape and intensity among the pinkish-red and dark-red spinels, it’s difficult to study the color contributions from fluorescence through the optical spectra. More discussions about fluorescence in spinel will be presented in the following.

### EEMs fluorescence spectra

**Figure 2:**
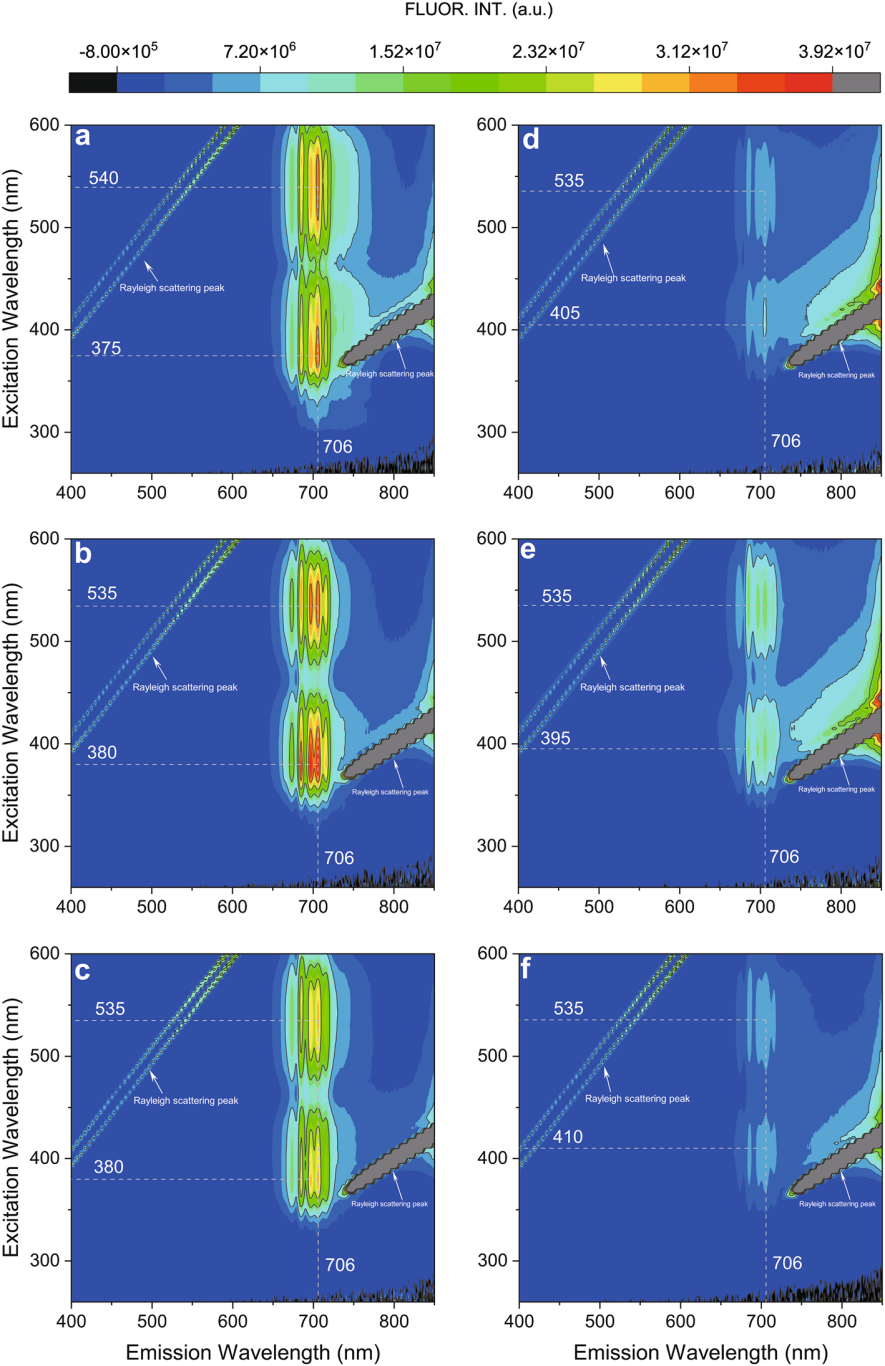
EEMs fluorescence spectra of the present spinels. Figure (**a**)–(**c**) on the left are the pinkish-red spinels (SPI-1 to SPI-3, respectively), and Figure (**d**)–(**f**) on the right are dark-red spinels (SPI-4 to SPI-6, respectively).

**Figure 3:**
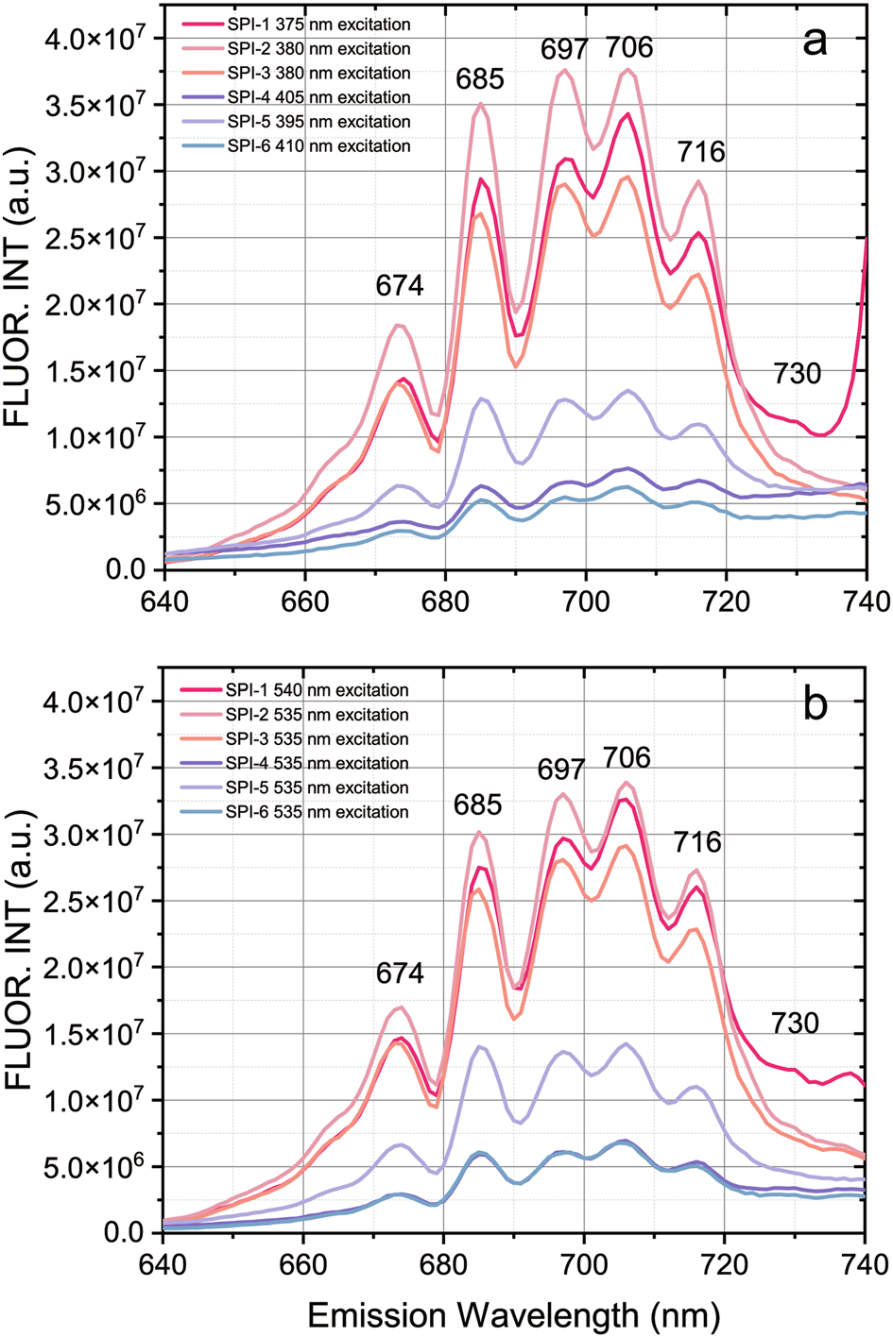
Emission spectra of the present spinels in specific excitation wavelength. (**a**) emission spectral curves excited by ~ 380 nm radiation. (**b**) Emission spectral curves excited by ~ 535 nm radiation.

The EEMs fluorescence spectra of the spinels are shown in Fig. 2. We found that there have no emission spectra under 400 nm, hence a 400 nm high-pass filter was utilized to reduce the Rayleigh scattering. The results indicated that the fluorescence spectra of the pinkish-red spinels are significantly stronger than that of the dark-red spinels. The fluorescence mapping demonstrates the pinkish-red spinels with live fluorescence should be excited by ~ 380 nm and ~ 535 nm radiations, both two radiations consequently produce 706 nm emission. On the contrary, the fluorescence mapping of the dark-red spinels is relatively much weaker than the former. It should be noted that the dark-red spinels could be excited by ~ 400 nm and ~ 535 nm radiations and produce 706 nm emissions as well. The strongest emission spectra of the spinel samples have a similar shape with peaks at 674 nm, 685 nm, 697 nm, 706 nm, 716 nm, and 730 nm (Fig. 3), they should be originated by optical transition ^2^*E*_*g*_ → ^4^*A*_2*g*_ in Cr^3+^ ion according to the previous research^28^. The emission intensity of the pinkish-red spinel excited by ~ 535 nm radiation is close to that excited by ~ 380 nm radiation. It proves that visible light could also excite the pinkish-red spinel to produce strong red fluorescence, as a result, the color of the pinkish-red spinel becomes more vivid and brighter. Considering this circumstance, the color contribution from fluorescence is essential for the color measurement of the pinkish-red spinel.

### Color analysis

**Figure 4:**
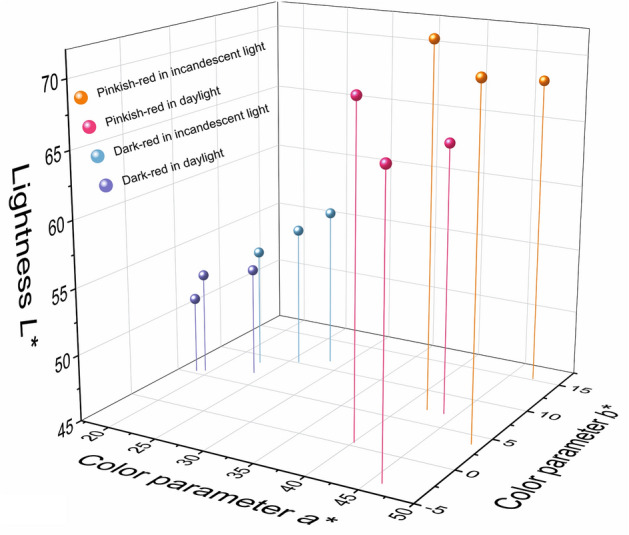
The spinel colors in daylight and incandescent light are plotted in the CIE1976 L^*^a^*^b^*^ uniform color space system.

To study the effect of a light source on spinel color, the color data of the spinel samples were measured by using an X-Rite Ci-7800 spectrophotometer with a background of Munsell N9 color chip for it is closed to gemstone common presentation in the jewelry industry. The whole experiment was conducted in the standard illumination box to eliminate the influence of the surrounding environment. The results are shown in Table 2. CIE standard illuminant D65 and illuminant A, which are commonly used to represent daylight and incandescent light, have different relative spectral power distributions as daylight with 6504 K color temperature has more energy in the blue-green radiation area while the energy of incandescent light with 2856 K color temperature concentrates more in the red zone^19^. It should be noted that the illuminants D65 and A don’t exist. But there are many different kinds of standard lamps were invented to represent these illuminants. The color data of the spinels in these two light sources were compared (Fig. 4). On the one hand, it showed the averages of the lightness, chroma, and hue angle of the pinkish-red spinels in daylight are 67.22, 43.56, and 1.80°, respectively. The averages of the same parameters for the dark-red spinels are 52.34, 21.48, and 16.44°, respectively. There are significant color differences with an average of 27.57 between pinkish-red and dark-red. These differences suggest the pinkish-red spinel is brighter and more vivid than the dark-red one, which corresponds to what we observe using our naked eyes. The same conclusion should be got when the spinels are illuminated by incandescent light. On the other hand, the results indicate there are large color differences with an average of 8.58 when the pinkish-red spinel are illuminated by daylight and incandescent light, while the dark-red spinels have a smaller value of 7.34 in the same circumstance even though the two kinds of spinels have similar optical absorptions. This difference is probably dues to the contribution of the strong red fluorescence in pinkish-red spinels, considering the D65 daylight has more energy in a short wavelength.

**Table 2:** Color data of the present spinel in daylight and incandescent light.

	Sample	SPI-01	SPI-02	SPI-03	SPI-04	SPI-05	SPI-06
Daylight (6504 K)	*X*	46.54	49.61	52.19	21.99	23.96	25.54
	*Y*	34.38	36.25	40.31	19.17	20.85	21.37
	*Z*	32.04	42.54	43.45	18.05	19.54	19.40
	L*	65.26	66.71	69.69	50.88	52.78	53.35
	a*	43.82	46.07	40.07	18.69	19.39	23.72
	b*	7.07	− 3.61	0.50	5.45	5.77	7.02
	C*	44.39	46.21	40.07	19.47	20.23	24.74
	*h*°	9.17	355.52	0.71	16.26	16.57	16.49
Incandescent light (2856 K)	*X*	60.89	66.83	66.69	30.12	33.18	35.71
	*Y*	38.42	42.12	44.33	22.06	23.73	25.10
	*Z*	9.81	13.90	13.67	6.26	6.53	6.73
	L*	68.33	70.95	72.45	54.09	55.82	57.17
	a*	47.24	48.88	42.12	22.73	25.89	28.39
	b*	15.22	3.72	7.11	8.80	10.18	11.36
	C*	49.63	49.02	42.72	24.37	27.82	30.58
	*h*°	17.86	4.35	9.58	21.16	21.46	21.81
460 nm UV filtrated							
Daylight (6504 K)	*X*	35.32	38.20	37.95	21.63	23.43	25.06
	*Y*	26.19	28.01	29.33	18.90	20.40	20.97
	*Z*	27.13	36.89	35.54	17.86	19.15	19.04
	L*	58.22	59.90	61.07	50.57	52.29	52.92
	a*	39.55	41.82	35.99	18.33	19.17	23.54
	b*	2.11	− 8.57	− 4.83	5.30	5.68	6.99
	C*	39.61	42.69	36.31	19.08	19.99	24.56
	*h*°	3.05	348.42	352.36	16.13	16.50	16.54
Incandescent light (2856 K)	*X*	60.40	64.63	65.12	29.15	32.05	34.46
	*Y*	38.11	40.69	43.36	21.41	23.04	24.34
	*Z*	9.78	13.43	13.42	6.15	6.37	6.59
	L*	68.10	69.96	71.80	53.39	55.11	56.43
	a*	47.11	48.45	41.57	22.19	25.12	27.55
	b*	14.96	3.66	6.90	8.21	9.88	10.89
	C*	49.43	48.59	42.14	23.66	26.99	29.62
	*h*°	17.62	4.32	9.42	20.30	21.47	21.57

As the discussions in the EEMs fluorescence spectra, there are two major excitement areas in natural Cr-containing spinel. To study the color contributions from the fluorescence in spinel, a 460 nm UV filter was utilized to isolate the influence of the radiations whose wavelengths are lower than 460 nm. The well-known tristimulus values of color could be calculated according to CIE (1931) or other works such as Nassau^20^. Similarly, the tristimulus values of the spinel filtrated color could be calculated by:

1 $${X}_{UV filtrated}=\frac{{\sum }_{380}^{460}S\left(\lambda \right) \overline{x }\left(\lambda \right) \Delta \lambda T\%\left(\lambda \right)+ {\sum }_{460}^{780}S\left(\lambda \right) \overline{x }\left(\lambda \right) \Delta \lambda {T\%\left(\lambda \right)}_{UV filtrated}}{{\sum }_{380}^{780}S\left(\lambda \right) \overline{y }\left(\lambda \right) \Delta \lambda }$$

2 $${Y}_{UV filtrated}=\frac{{\sum }_{380}^{460}S\left(\lambda \right) \overline{y }\left(\lambda \right) \Delta \lambda T\%\left(\lambda \right)+ {\sum }_{460}^{780}S\left(\lambda \right)\overline{ y }\left(\lambda \right) \Delta \lambda {T\%\left(\lambda \right)}_{UV filtrated}}{{\sum }_{380}^{780}S\left(\lambda \right) \overline{y }\left(\lambda \right) \Delta \lambda }$$

3 $${Z}_{UV filtrated}=\frac{{\sum }_{380}^{460}S\left(\lambda \right) \overline{Z }\left(\lambda \right) \Delta \lambda T\%\left(\lambda \right)+ {\sum }_{460}^{780}S\left(\lambda \right) \overline{Z }\left(\lambda \right) \Delta \lambda { T\%\left(\lambda \right)}_{UV filtrated}}{{\sum }_{380}^{780}S\left(\lambda \right) \overline{y }\left(\lambda \right) \Delta \lambda }$$

wherein S(λ) is the relative spectral power distribution of the light source,${\overline{\text{x}}}$(λ), ${\overline{\text{y}}}$(λ), and ${\overline{\text{z}}}$(λ) are the CIE color-matching functions for the Standard Observer at the wavelength λ, “${\sum }_{380}^{780}\Delta \lambda$” represents Riemann summation over the visible portion of the electromagnetic spectrum with λ in units of nanometers, T%(λ) is the percentage of light transmitted through the material at a specific wavelength λ.

**Figure 5:**
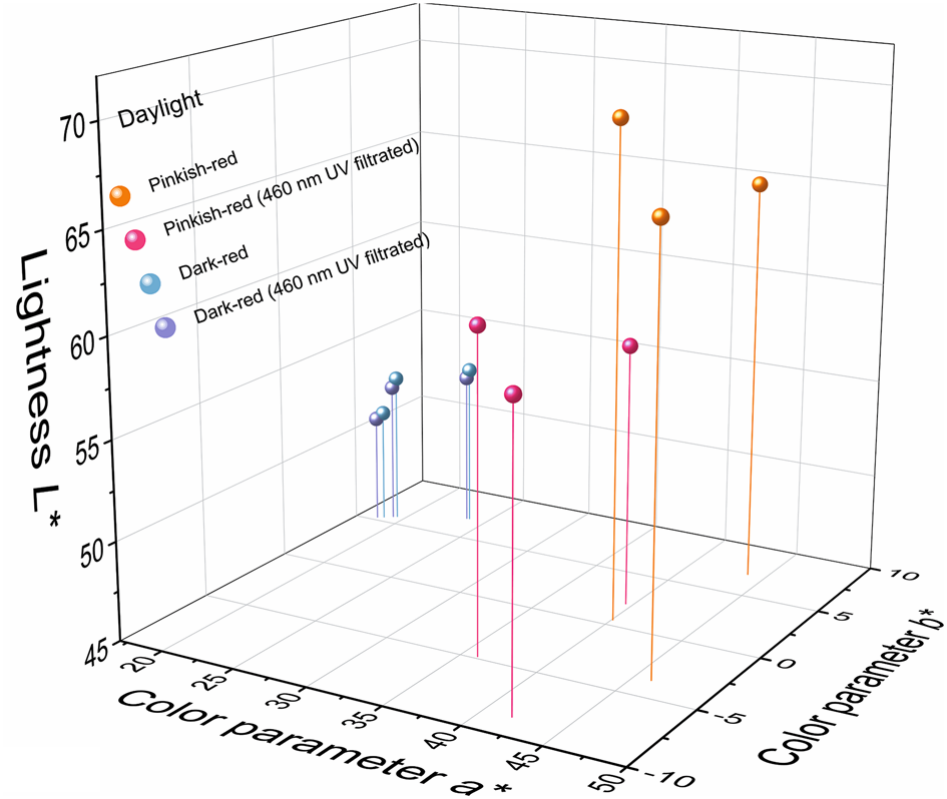
The spinel colors were filtrated by 420 nm in daylight.

**Figure 6:**
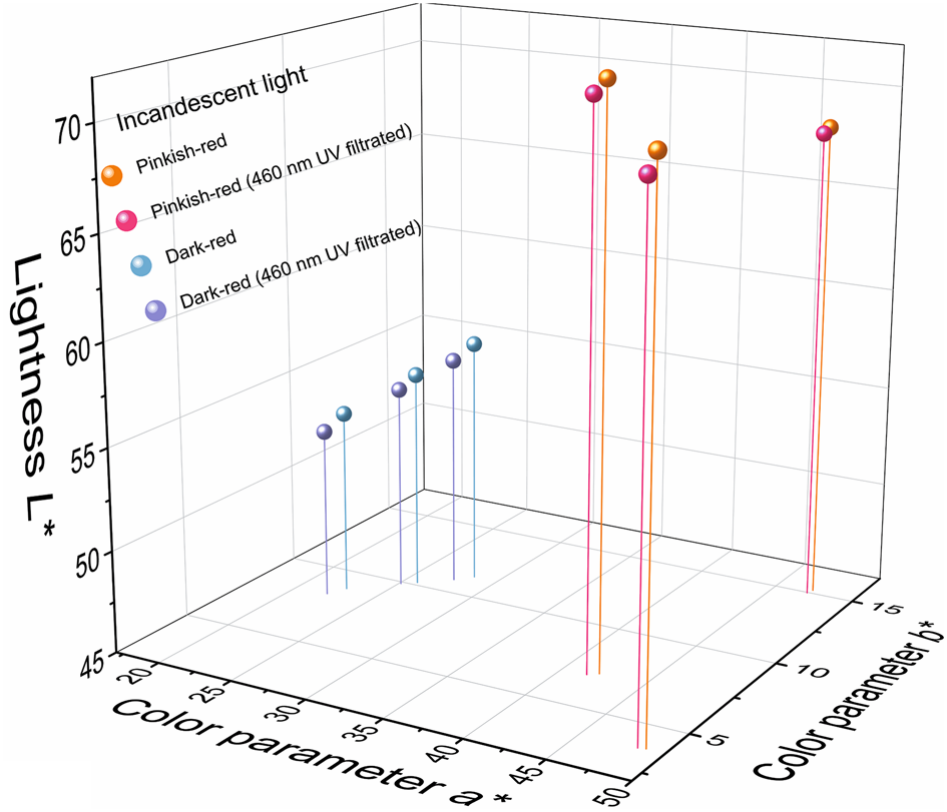
The spinel colors are filtrated by 420 nm in incandescent light.

The results are presented in Table 2 and the color data were plotted in the color space as shown in Fig. 5 (daylight) and Fig. 6 (incandescent light). For pinkish-red spinels, the color differences before and after UV filtration are significant with an average of 9.99, while the same parameter for dark-red spinels is only 0.50. The lightness, chroma, and hue angle of the pinkish-red went down with averages of 59.73, 39.54, and 354.61° (converted from − 5.39°). It illustrates that the fluorescence produced by < 460 nm radiation contributes more than 10% lightness and chroma in Cr-containing spinel under daylight. On the contrary, when the spinels are illuminated by incandescent light, the color differences before and after UV filtration are very small with averages of 0.78 for pinkish-red and 1.12 for dark-red, which may be caused by the low power in the blue-green area in incandescent light. However, the color contribution from ~ 535 nm excitation is unclear due to the technical limitation, it will be a subject for further study.

Despite the influence of light sources, the background color has great effects on the color of crystals due to their high transparency. Hence, it is important to consider the background color when we measure or calculate the color of transparent minerals. It determines whether the color of minerals will be described precisely, even more, it contributes to further studies in the applications of deep learning for mineral sorting and identification, etc. In this research, nine pieces of Munsell neutral value grayscales were used as background behind the spinels to simulate spinel colors in different grayscale environments.

The lightness *L*_b_^*^ of the Munsell neutral value gray scales have a power function relation with their luminance factors *Y*_*b*_, which is as follows:

4 $${L}_{b}^{*}=116{Y}_{b}^{1/3}-16$$

**Figure 7:**
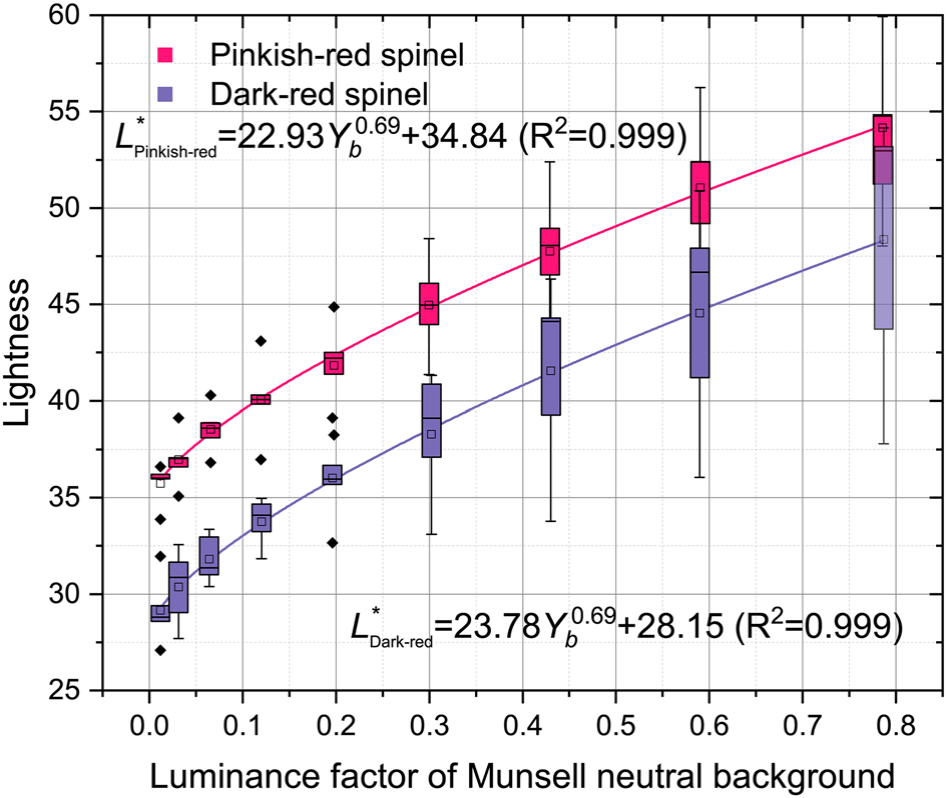
The lightness of spinel increases with the luminance factor (*Y*_*b*_) of the Munsell neutral background in the power function.

**Figure 8:**
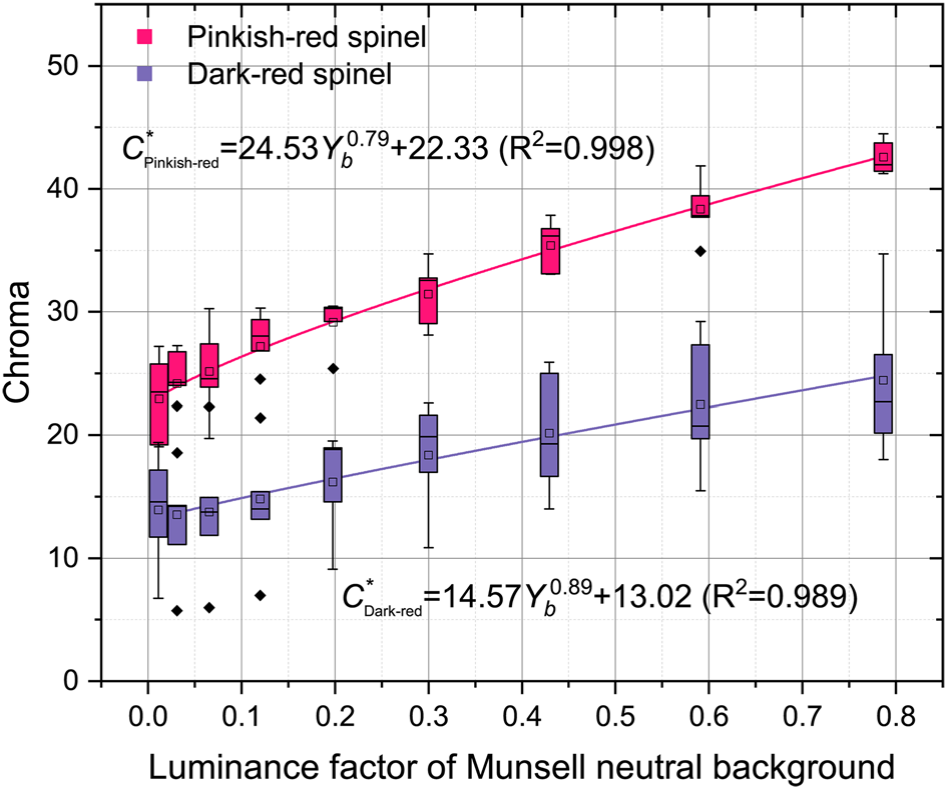
The Chroma of spinel increases with the luminance factor (*Y*_*b*_) of the Munsell neutral background in power function.

To study the influence of background on spinel color, nine different gray scales of neutral backgrounds and D65 light source were adopted. The results indicated that the lightness (Fig. 7) and chroma (Fig. 8) of all spinel colors increase with the luminance factor of Munsell neutral background in power function. For pinkish-red spinels and the dark-red ones, there are power function relationships between their lightness L^*^ and background luminance factor *Y*_*b*_, which are as follows:

5 $${L}_{Pinkish{\text{-}}red}^{*}=22.39{Y}_{b}^{0.69}+34.84 ({R}^{2}=0.999)$$

6 $${L}_{Dark{\text{-}}red}^{*}=23.78{Y}_{b}^{0.69}+28.15 ({R}^{2}=0.999)$$

Similar relations also appear between their chroma C^*^ and background luminance factor:

7 $${C}_{Pinkish{\text{-}}red}^{*}=24.53{Y}_{b}^{0.79}+22.33 \left({R}^{2}=0.998\right)$$

8 $${C}_{Dark{\text{-}}red}^{*}=14.57{Y}_{b}^{0.89}+13.02 ({R}^{2}=0.989)$$

Despite the lightness and chroma, the hue angle of the spinel is hardly affected by the reflections of the neutral gray backgrounds.

In general, the spinels' lightness and chroma grow with the decrease of the background grayscale. The differentiation of spinel colors in dark conditions is lower than that in a bright environment. The color of the spinel could be predicted by the equations as long as the luminance factor of the background is provided, or the system will automatically recognize the spinel by matching its color and the reflection of the background.

## Conclusion

The higher value of Cr/Fe makes pinkish-red spinel has a much stronger red fluorescence effect than dark-red spinel. The two narrow absorption bands at ~ 25,500 cm^−1^ (with a broad absorption band at ~ 24,100 cm^−1^) and ~ 18,570 cm^−1^ in the optical absorption spectrum are assigned to spin-allowed electronic *d-d* transitions ^4^*A*_2*g*_ → ^4^*T*_1*g*_(F) and ^4^*A*_2*g*_ → ^4^*T*_2*g*_(F) in Cr^3+^ at the *M* site. The EEMs spectra of the pinkish-red spinels show twin emissions at 706 nm excited both by ~ 380 nm and ~ 535 nm radiations, which is the key to the bright neon red color in pinkish-red spinel. The colorimetry study suggests the strong red fluorescence produced by < 460 nm radiation contributes more than 10% lightness and chroma in pinkish-red spinel under daylight. The lightness and the chroma of the spinels grow with the decrease of background grayscale. The differentiation of spinel colors in dark conditions is lower than that in a bright environment.

## Material and methods

### Samples

**Figure 9:**
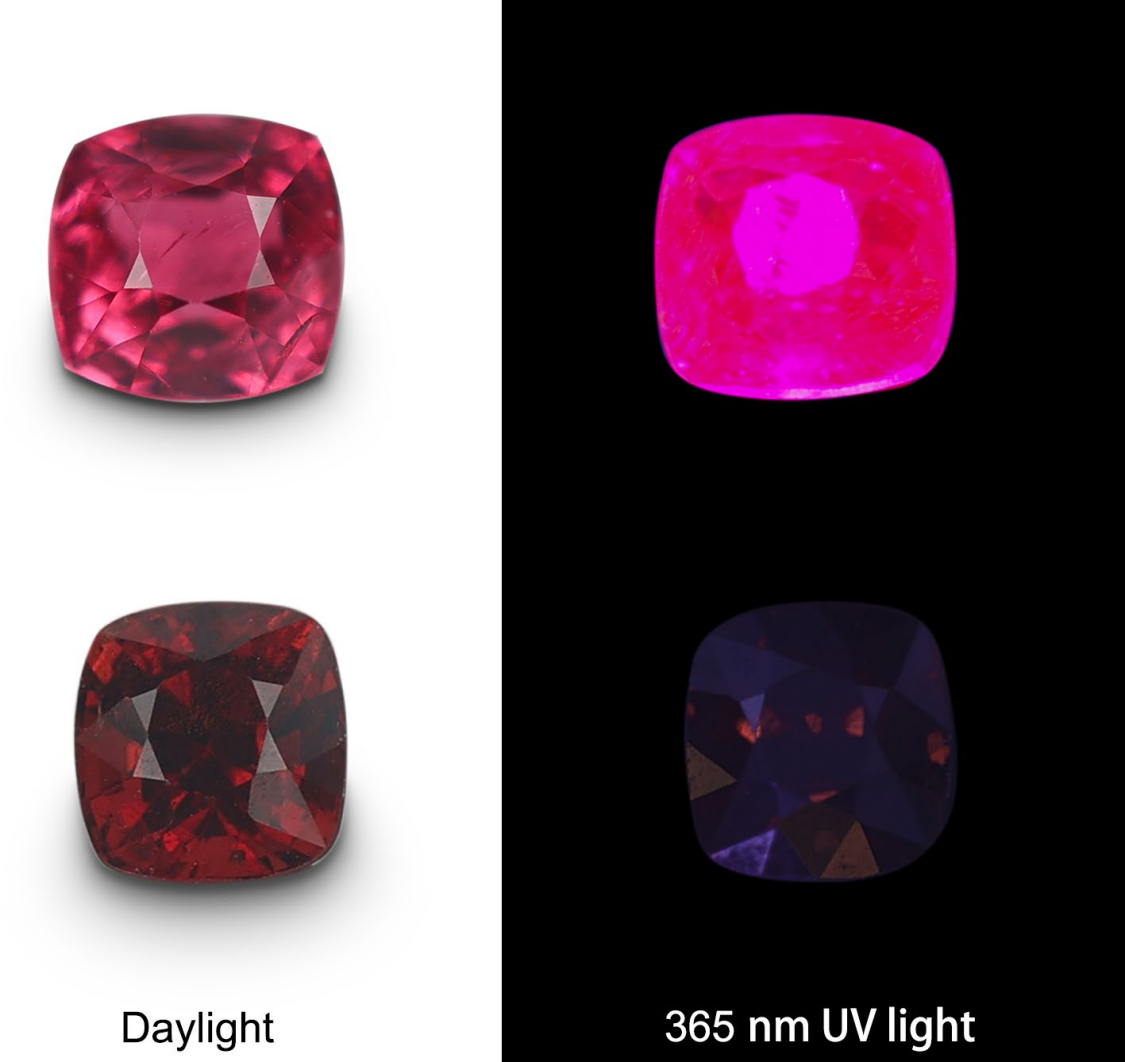
Pinkish-red and dark-red faceted spinel under daylight and 365 nm UV light.

Six natural spinel crystals from Myanmar with high transparency, including three pinkish-red spinels (from SPI-1 to SPI-3) which have a strong red fluorescence effect under long wavelength UV light (365 nm), and three dark-red spinels (from SPI-4 to SPI-6) which have no obvious fluorescence (Fig. 9), were cut into wafers (length 4.128–4.390 mm, width 3.698–4.411 mm, height 1.322–1.407 mm). The double parallel sides of the wafer ensure the accuracy of color measuring.

### Electron microprobe (EMP) analysis

Major element compositions of minerals were analyzed using a JXA-8230 electron microprobe at the Beijing ZKKY GeoAnalysis Laboratory Co., Ltd. The operating conditions were an accelerating voltage of 15 kV for silicate and oxide, 20 kV for sulfide, a beam current of 20 nA, and a beam size of 5 μm. Natural minerals and synthetic oxides were used as standards. Matrix corrections were carried out using the ZAF correction program supplied by the manufacturer.

### LA-ICP-MS analysis

Trace element compositions were determined using an ESI NWR 193 nm excimer laser coupled with an Agilent 7500 ICP-MS at the Beijing ZKKY GeoAnalysis Laboratory Co., Ltd. Analyses were performed with a beam diameter of 35 μm and a repletion rate of 8 Hz. Helium, was applied as a carrier gas, was mixed with Argon via a T-connector before entering the ICP-MS. Each analysis incorporated a background acquisition (from a gas blank) of 20 s followed by 45 s of data acquisition from the sample. The international glass NIST 610, was used as the primary standard for trace element composition, of which value is available in the GeoReM database (http://georem.mpchmainz.gwdg.de/). An Excel-based software ICPMSDataCal^29^, was adopted to perform off-line correction using ^27^Al as an internal standard element.

### Optical absorption spectroscopy

Optical absorption spectra were measured in the 300 to 800 nm range using a UV-3600 UV–vis spectrophotometer at room temperature and a 0.5 nm spectral resolution at a scan speed of 400 nm/min. The detector conversion wavelength is 850 nm and the grating conversion wavelength is 900 nm with S/R shift.

### Excitation-emission matrices (EEMs) fluorescence spectroscopy

The EEMs fluorescence spectroscopy was widely used in material science^30^ and gemology^11^. All spectra were recorded at room temperature using a HORIBA Scientific Nanolog FL3-2iHR fluorescence spectrometer at Peking University. EEMs spectra were collected with a 1500 nm/min scan speed. The excitation wavelengths varied from 260 to 600 nm, with a step size of 5 nm. The emission wavelengths varied from 270 to 850 nm, with a step size of 1 nm. The Rayleigh scattering was subdued by using a 400 nm high-pass filter.

### CIE1976 L^*^a^*^b^*^ uniform color space system

The color system is composed of colorimetric coordinates a^*^, b^*^, and lightness L^*^. Thereinto, + a^*^ represents red while –a^*^ represents green, and + b^*^ represents yellow while –b^*^ represents blue. Chroma *C*_ab_^*^ and hue angle *h*_*ab*_^*°*^ should be calculated by a^*^ and b^*^:

9 $${C}_{ab}^{*}={\left[{\left({a}^{*}\right)}^{2}+{\left({b}^{*}\right)}^{2}\right]}^{1/2}$$

10 $${h}_{ab}^{^\circ }=arctan\frac{{b}^{*}}{{a}^{*}}$$

To calculate the color difference of spinels, CIE DE2000 (Δ*E*_*00*_) formula which was recommended by Commission Internationale de L'Eclairage (CIE) was applied. Compared with the CIE *LAB* (Δ*E*_ab_^*^) formula which lacks visual uniformity^31^, Δ*E*_*00*_ calculates color difference more precisely in the green area, it’s an improved version of Δ*E*_ab_^*^. The formula is as follows:

11 $${\Delta E}_{00}={\left[{\left(\frac{{\Delta L}^{^{\prime}}}{{k}_{L}{S}_{L}}\right)}^{2}+{\left(\frac{{\Delta C}^{^{\prime}}}{{k}_{C}{S}_{C}}\right)}^{2}+{\left(\frac{{\Delta H}^{^{\prime}}}{{k}_{H}{S}_{H}}\right)}^{2}+{R}_{T}{\left(\frac{{\Delta C}^{^{\prime}}}{{k}_{C}{S}_{C}}\right)}^{2}\left(\frac{{\Delta H}^{^{\prime}}}{{k}_{H}{S}_{H}}\right)\right]}^{1/2}$$

Δ*L′*, Δ*C′,* and Δ*H′* are lightness difference, chroma difference, and hue angle difference of a pair of color data, respectively. *R*_*T*_ is a function to reduce the interaction between chroma and hue in the blue area. *S*_*L*_, *S*_*C,*_ and *S*_*H*_ are functions to calibrate the absence of visual uniformity of the CIE *LAB* formula. *K*_*L*_, *K*_*C,*_ and *K*_*H*_ are correction parameters of the environment. There are two widely used combinations of (*K*_*L*_, *K*_*C*_, and *K*_*H*_): CIE DE2000 (1:1:1)^32^ and CIE DE2000 (2:1:1)^33^. CIE DE2000 (1:1:1) was applied in this study because it has a better perceptibility when evaluating color differences.

### Colorimetric analysis

The spinel colors were acquired by an X-Rite Ci-7800 sphere benchtop spectrophotometer, measuring from 360 to 780 nm in a 5 nm wavelength interval in transmission mode. It measured for 4 s and used pulsed-xenon D65 calibrated lamp as illumination. The 460 nm UV filter was employed to study the color contributions from fluorescence in spinel. The software X-Rite Color iQC was utilized to analyze and output color data. Munsell neutral value gray scales chart is a grayscale fan deck with values of 0.5/ to 9.5/, in quarter-step intervals. It is frequently used for instrumental calibration, imaging testing, or as reflection standards. Nine Munsell neutral value gray scales (glossy edition) with different gray scales or luminance factors (*Y*_b_) including N1 (*Y*_b_ = 1.210%), N2 (*Y*_b_ = 3.126%), N3 (*Y*_b_ = 6.555%), N4 (*Y*_b_ = 12.000%), N5 (*Y*_b_ = 19.770%), N6 (*Y*_b_ = 30.050%), N7 (*Y*_b_ = 43.060%), N8 (*Y*_b_ = 59.100%), and N9 (*Y*_b_ = 78.660%) were utilized as backgrounds in this experiment. A standard illumination box was adopted in the experiment.

## Data Availability

The dataset and the samples for this study is available from the corresponding author upon reasonable request.
